# Pyrrolo[2,3‐*d*]pyrimidine (7‐deazapurine) as a privileged scaffold in design of antitumor and antiviral nucleosides

**DOI:** 10.1002/med.21465

**Published:** 2017-08-23

**Authors:** Pavla Perlíková, Michal Hocek

**Affiliations:** ^1^ Institute of Organic Chemistry and Biochemistry Czech Academy of Sciences CZ‐16610 Prague 6 Czech Republic; ^2^ Department of Organic Chemistry Faculty of Science Charles University in Prague CZ‐12843 Prague 2 Czech Republic

**Keywords:** antivirals, cytostatics, deazapurines, nucleosides, nucleotides

## Abstract

7‐Deazapurine (pyrrolo[2,3‐*d*]pyrimidine) nucleosides are important analogues of biogenic purine nucleosides with diverse biological activities. Replacement of the N7 atom with a carbon atom makes the five‐membered ring more electron rich and brings a possibility of attaching additional substituents at the C7 position. This often leads to derivatives with increased base‐pairing in DNA or RNA or better binding to enzymes. Several types of 7‐deazapurine nucleosides with potent cytostatic or cytotoxic effects have been identified. The most promising are 7‐hetaryl‐7‐deazaadenosines, which are activated in cancer cells by phosphorylation and get incorporated both to RNA (causing inhibition of proteosynthesis) and to DNA (causing DNA damage). Mechanism of action of other types of cytostatic nucleosides, 6‐hetaryl‐7‐deazapurine and thieno‐fused deazapurine ribonucleosides, is not yet known. Many 7‐deazaadenosine derivatives are potent inhibitors of adenosine kinases. Many types of sugar‐modified derivatives of 7‐deazapurine nucleosides are also strong antivirals. Most important are 2′‐*C*‐methylribo‐ or 2′‐*C*‐methyl‐2′‐fluororibonucleosides with anti‐HCV activities (several compounds underwent clinical trials). Some underexplored areas of potential interest are also outlined.

## 1 INTRODUCTION

Pyrrolo[2,3‐*d*]pyrimidine (7‐deazapurine) nucleosides is a class of compounds closely related to purine nucleosides. The shape of pyrrolo[2,3‐*d*]pyrimidine resembles purine and therefore 7‐deazapurine nucleosides can substitute purine nucleosides in DNA and RNA [for the reference to natural purines, we will use the (deaza)purine nomenclature and numbering instead of the IUPAC preferred pyrrolo[2,3‐*d*]pyrimidine nomenclature].

**Figure 1:**
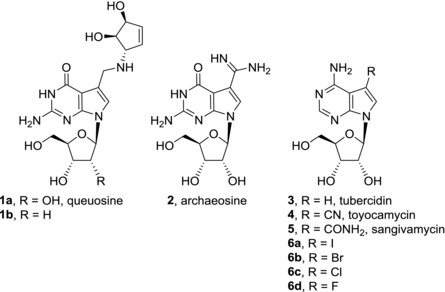
Naturally occurring deazapurine nucleosides (**1**–**5**) and related compounds

Some 7‐deazapurine nucleosides even occur in nature—both as nucleosides and as components of nucleic acids. Queuosine (**1a**)^1^, ^2^, ^3^ and archaeosine (**2**)^4^ are examples of 7‐deazapurine nucleosides isolated from tRNA of prokaryotic and eukaryotic organisms and archaea, respectively. Recently, the presence of 2′‐deoxyqueuosine (**1b**) was also discovered in bacterial DNA^5^ (Fig. 1). Several nucleoside antibiotics contain pyrrolo[2,3‐*d*]pyrimidine nucleobase and their biological activities will be mentioned later in the text. The biosynthetic pathway to 7‐deazapurines uses GTP as a precursor. More details about 7‐deazapurine biosynthesis can be found in a detailed review.^6^

Replacement of the N7 nitrogen by carbon changes the electronic properties of the five‐membered ring, making it more electron‐rich and thus (at least in theory) more prone to cation–π or π–π interactions. Also, the presence of this additional C7 carbon gives a suitable position for attachment of substituents which (in DNA or RNA) point out to the major groove. It has been repeatedly shown that 7‐substituted 7‐deazapurine nucleoside triphosphates are good substrates for DNA or RNA polymerases and DNA containing 7‐substituted 7‐deazapurines forms stable duplexes (some 7‐alkynyl‐ or 7‐aryl‐7‐deazapurines nucleotides are even better substrates for polymerases than natural dATP or dGTP^7^, ^8^ and in DNA they stabilize duplexes^9^). For applications in medicinal chemistry, the position C7 offers a possibility for functionalization and many 7‐substituted 7‐deazapurine nucleosides display important biological activities which are summarized and discussed in this review. It should be noted that a general review on syntheses and biological activities of pyrrolopyrimidines was published recently,^10^ but it did not specifically cover nucleosides and completely missed many important classes of relevant compounds. The synthetic approaches to 7‐deazapurine nucleosides are covered in the above‐mentioned review^10^ or in a recent book.^11^ This review focuses on recent (2000–2016) advances in medicinal chemistry of 7‐deazapurine nucleosides and is based on SciFinder, Reaxys, and Web of Knowledge searches.

## 2 CYTOTOXIC NUCLEOSIDES

### 2.1 Natural compounds

Cytotoxic activities of naturally occurring 7‐deazapurine nucleosides were discovered already in 1960s. Tubercidin (**3**), toyocamycin (**4**), and sangivamycin (**5**) were isolated from *Streptomyces* cultures. All of them show potent cytotoxicity against cancer cell lines^12^, ^13^, ^14^, ^15^ but despite their structural similarities, their modes of action are different (IC_50_ values for compounds mentioned in this section are listed in Table 1). Tubercidin (**3**), toyocamycin (**4**), and sangivamycin (**5**) are phosphorylated by cellular kinases to their mono‐, di‐, and triphosphate forms and the resulting nucleotides are then incorporated into RNA and DNA which causes damage to nucleic acid functions.^16^, ^17^, ^18^, ^19^, ^20^ Furthermore, tubercidin (**3**) impairs numerous cellular processes such as pre‐mRNA processing and nuclear speckle formation,^21^ mitochondrial respiration, purine synthesis, rRNA processing, and methylation of tRNA.^22^ Tubercidin (**3**) is also a potent inhibitor of *S*‐adenosylhomocysteine hydrolase.^23^ Toyocamycin (**4**) showed inhibition of phosphatidylinositol kinase^24^, inhibitory effect on rRNA synthesis, and maturation.^25^, ^26^ In a more recent study, toyocamycin (**4**) was identified as a potent inhibitor of induced XBP1 (X‐box binding protein 1) mRNA cleavage. IRE1α‐XBP1 pathway is a key component of endoplasmic reticulum stress response. IRE1α‐XBP1 inhibition by toyocamycin (**4**) leads to apoptosis in endoplasmic reticulum‐stressed tumors and multiple myeloma.^27^ On the other hand, cytotoxic effect of sangivamycin (**5**) is mainly caused through potent and selective inhibition of protein kinase C.^28^ Recently, the mechanism of action of sangivamycin (**5**) in primary effusion lymphoma cells was studied showing that sangivamycin (**5**) acts through inhibition of Erk and Akt signaling in these cells.^15^ Sangivamycin (**5**) is also able to bind heat‐shock protein 70 (HSP70) (K_D_ = 3.3 μM) which is a molecular chaperone with a proposed importance in oncology.^29^ Despite the attention paid to the naturally occurring 7‐deazapurine nucleosides, none of the compounds proceeded to clinical use.

**Table 1:** Cytotoxic activities of 7‐deazapurine nucleosides

Compound	IC_50_ [μM] (cell line)	Ref.
Natural compounds		
Tubercidin (**3**)	0.001 (A549)	12
Toyocamycin (**4**)	0.012 (HTB‐81)	13
Sangivamycin (**5**)	0.006 (A549)	14
7‐Substituted 7‐deazapurine ribonucleosides		
7‐Iodotubercidin (**6a**)	2.6^a^ (HeLa)	35
7‐Bromotubercidin (**6b**)	2.9^a^ (HeLa)	35
7‐Chlorotubercidin (**6c**)	13.3^a^ (HeLa)	35
7‐Fluorotubercidin (**6d**)	1 (L‐1210)	36
AB61 (**7a**)	0.01 (A549); 0.00036 (CCRF‐CEM)	42
**7b**	0.35 (A549); 0.10 (CCRF‐CEM); 0.003 (HCT15)	12
**7c**	>20 (A549); > 10 (CCRF‐CEM)	12
**7d**	0.701 (A549); >10 (CCRF‐CEM); 0.152 (Du145)	12
**8**	43.76 (A549); 64.44 (CCRF‐CEM)	41
**9a**	0.03 (A549); 0.02 (CCRF‐CEM);	44
**9b**	0.05 (A549); 0.05 (CCRF‐CEM); 0.01 (HCT116p53‐/‐)	44
**9c**	2.57 (A549); 0.18 (CCRF‐CEM);	44
**9d**	4.60 (A549); 2.91 (CCRF‐CEM); 0.15 (K562)	44
**9e**	0.11 (A549); 0.04 (CCRF‐CEM)	44
**10a‐c**	>20 (A549); >20 (CCRF‐CEM)	44
**11a**	0.01 (CCRF‐CEM); 0.001 (Hs578)	12
**11b**	0.14 (A549); >20 (CCRF‐CEM); 0.03 (HCT116)	44
**11c**	0.39 (A549); 0.09 (CCRF‐CEM)	44
**11d**	19.2 (A549); 5.60 (CCRF‐CEM); 0.11 (K562‐TAX)	44
**12a**	0.4 (HeLa)	45
**12b**	0.2 (HeLa)	45
Sugar‐modified 7‐substituted 7‐deazapurine nucleosides		
**13a**	4.25 (CCRF‐CEM)	46
**13b**	NA^b^ (CCRF‐CEM)	46
**13c**	NA (CCRF‐CEM)	46
**13d**	7.50 (CCRF‐CEM)	46
**13e**	>10 (CCRF‐CEM)	47
**13f**	3.44 (CCRF‐CEM)	47
**14a**	6.64 (CCRF‐CEM)	46
**14b**	6.08 (NCI‐H23)	46
**14c**	NA (CCRF‐CEM)	46
**14d**	8.49 (Hs578)	46
**14e**	>10 (CCRF‐CEM)	47
**14f**	6.63 (CCRF‐CEM)	47
**15**	0.18 (CCRF‐CEM); 0.002 (Du145)	46
**16a**	0.64 (CCRF‐CEM); 0.15 (HepG2)	47
**16b**	0.42 (CCRF‐CEM); 0.35 (HepG2)	47
**17**	3.5 (CCRF‐CEM)	48
**18**	7.13 (A549); 16.63 (CCRF‐CEM)	49
8‐Substituted deazapurine nucleosides		
ARC (**21a**)	0.97 (SW620); 0.49 (DM366); 0.20 (SK‐N‐AS)	54, 55, 56
Xylocydine (**22a**)	>50 (A549)	64
JRS‐15 (**23b**)	12.42 (HepG2)	64
**24**	2.6 (PA‐1)	70
6‐Substituted 7‐deazapurine ribonucleosides and pronucleotides		
**25a**	0.088 (A549); 0.31 (CCRF‐CEM); 0.007 (Du145)	71
**25b**	0.045 (A549); 0.29 (CCRF‐CEM); 0.009 (Du145)	71
**25c**	>10 (A549); 4.0 (CCRF‐CEM); 1.1 (Du145)	71
**26a**	0.11 (A549); 1.4 (CCRF‐CEM); 0.005 (Du145)	71
**26b**	0.061 (A549); 0.27 (CCRF‐CEM); 0.009 (Du145)	71
**27a**	>10 (A549); 3.8 (CCRF‐CEM)	71
**27b**	0.38 (A549); 2.7 (CCRF‐CEM); 0.013 (Du145)	71
**28a**	2.00 (CCRF‐CEM); 0.017 (HCT116)	72
**28b**	18.60 (CCRF‐CEM); 0.011 (Du145)	72
**28c**	>10 (CCRF‐CEM)	72
**29a**	17.67 (CCRF‐CEM); 0.007 (Du145)	72
**29b**	>10 (CCRF‐CEM); 0.022 (Du145)	72
**30a**	3.44 (CCRF‐CEM); 1.03 (HCT116)	73
**30b**	3.34 (CCRF‐CEM); 1.02 (HeLa S3)	73
**30c**	1.13 (CCRF‐CEM); 0.58 (HeLa S3)	73
**31a**	8.47 (CCRF‐CEM); 1.41 (HepG2)	73
**31b**	>10 (CCRF‐CEM); 1.53 (HepG2)	73
**31c**	2.09 (CCRF‐CEM); 0.68 (HCT116)	73
**32a**	29.4 (A549); 2.22 (CCRF‐CEM)	74
**32b–d**	>150 (A549); > 150 (CCRF‐CEM)	74
Sugar‐modified 6‐substituted 7‐deazapurine nucleosides		
**33a‐e**	NA (CCRF‐CEM)	75, 76
**34**	NA (A549); NA (CCRF‐CEM)	49
Other 7‐deazapurine nucleosides		
**35a**	0.03 (A549); 0.01 (CCRF‐CEM)	44
**35b–e**	>150 (A549); >150 (CCRF‐CEM)	74
**36a**	0.028 (HepG2); 0.090 (HeLa S3)	77
**36b–h**	>50 (HepG2); >50 (HeLa S3)	77
**37**	115 (CCRF‐CEM); 1.8 (HeLa)	78
**38**	36.20 (A549)	79
Fused 7‐deazapurine nucleosides		
**41a**	7.9 (CCRF‐CEM)	91
**41b**	20.2 (CCRF‐CEM); 2.0 (L‐1210)	91, 92
**42**	0.175 (HepG2)	93
**43a**	18 (CCRF‐CEM); 11 (HeLa S3)	94
**43b**	12 (CCRF‐CEM)	94
**44a**	>50 (A549); 47.62 (CCRF‐CEM)	95
**44b**	11.9 (A549); 6.22 (CCRF‐CEM); 2.5 (U2OS)	95
**44c**	0.21 (A549); 0.02 (CCRF‐CEM)	95
**44d**	0.22 (A549); 0.13 (CCRF‐CEM); 0.11 (HL60)	95
**44e**	0.66 (A549); 0.027 (CCRF‐CEM)	95
**45a**	>50 (A549); >50 (CCRF‐CEM)	95
**45b**	30.11 (A549); 0.3 (CCRF‐CEM)	95
**45c**	0.72 (A549); 0.03 (CCRF‐CEM)	95
**45d**	1.23 (A549); 0.22 (CCRF‐CEM)	95
**45e**	0.80 (A549); 0.20 (CCRF‐CEM)	95

a minimal cytotoxic concentration; ^b^NA = inactive. Cell lines mentioned in the table: A549, human lung carcinoma; CCRF‐CEM, human lymphoblastic leukemia; DM366, human melanoma; Du145, human prostate carcinoma; HCT116, human colorectal carcinoma; HCT116p53−/−, human colorectal carcinoma, p53 mutated; HCT15, human colorectal adenocarcinoma; HeLa, human cervical adenocarcinoma; HeLa S3, human cervical adenocarcinoma, HepG2, human hepatocellular carcinoma; HL60, human acute promyelocytic leukemia; Hs578, human breast carcinoma; HTB‐81, human prostate carcinoma; K562, human chronic myelogenous leukemia; K562‐TAX, human colorectal carcinoma, taxol‐resistant; L‐1210, mouse lymphocytic leukemia; NCI‐H23, human lung adenocarcinoma; PA‐1, human teratocarcinoma; SK‐N‐AS, human neuroblastoma; SW620, human colon adenocarcinoma; U2OS, human osteosarcoma.

### 2.2 Synthetic nucleosides with C7 and C8 substituents

**Figure 2:**
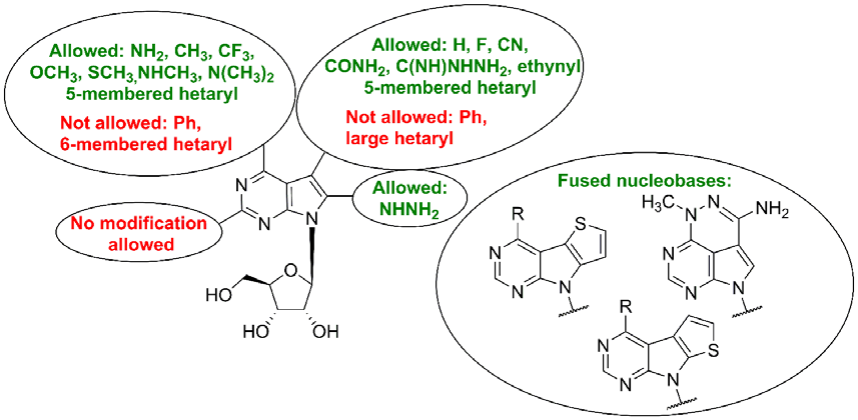
Structure‐activity relationship among cytotoxic 7‐deazapurine ribonucleosides

First structure‐activity studies among 7‐deazapurine nucleosides focused on modifications of the sugar moiety of the nucleosides. Even though many sugar‐modified analogs of naturally occurring 7‐deazapurine nucleosides (e.g. deoxynucleosides, *ara*‐, and *xylo*‐diastereomers) were synthesized, modification of the sugar moiety typically led to a decrease in cytotoxic activity.^13^, ^14^, ^30^, ^31^, ^32^, ^33^ Therefore, nucleobase‐modified derivatives and compounds combining nucleobase and sugar modifications attracted more attention. 7‐Halogenated tubercidins (**6a–d**)^34^ differ in their cytotoxic activity depending on the nature of the halogen (Fig. 2). 7‐Iodo‐, bromo‐, and chlorotubercidin (**6a–c**) are less cytotoxic compared with tubercidin (**3**).^35^ On the other hand, 7‐fluorotubercidin (**6d**) showed superior cytotoxicity over tubercidin (**3**) and improved selectivity towards leukemic cells^36^ but its mechanism of action has not been studied. 7‐Iodotubercidin (**6a**) acts mainly as a potent inhibitor of adenosine kinase.^37^ For example, it showed promising submicromolar cytotoxicity in canine osteosarcoma cell lines which show dysregulation of kinase activity.^38^ 7‐Iodotubercidin (**6a**) is also a p53 activator, and generates DNA damage and G2 cell cycle arrest through its incorporation into DNA.^39^ On the contrary, the cytotoxic effect of 7‐bromotubercidin (**6b**) is caused by incorporation of its triphosphate form into cellular RNA and inhibition of RNA synthesis.^22^

**Figure 3:**
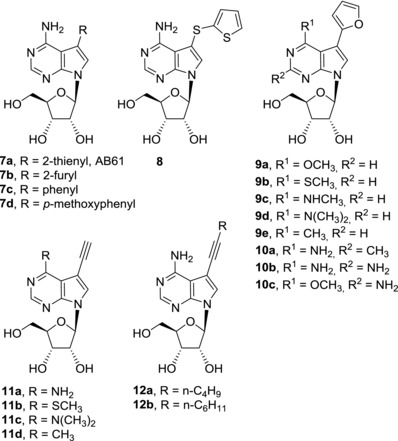
Cytotoxic C7‐substituted deazapurine ribonucleosides

In our laboratory, we developed a new group of potent nucleoside cytostatics by the attachment of heterocycles to C‐7 position of 7‐deazaadenosine^12^ (Fig. 3). It was shown that derivatives substituted with five‐membered heterocycles, i.e. thiophene or furan (**7a**‐**b**) exerted nanomolar in vitro cytotoxicity to broad panel of cancer and leukemia cell lines [similar to tubercidin (**3**)], while phenyl derivative **7c** is apparently too bulky and therefore inactive. Similar results were reported by Schram and Townsend in their pioneering study on synthesis of 7‐hetaryl‐7‐deazapurine ribonucleosides.^40^ Derivatives with five‐membered rings showed mild antileukemic activity and six‐membered ring derivatives were inactive, however IC_50_ values are not given in this study. Electronic properties of the substituents are also important for the activity. The presence of an electron‐donating *p*‐methoxy group on a phenyl substituent made compound **7d** active in submicromolar concentrations. Direct connection of thiophene and 7‐deazapurine moieties in **7a** (known as AB61) is also crucial for the cytotoxic activity, as its analogue **8** with the thienyl group attached via a sulfur atom showed a substantial drop of activity (IC_50_ > 20 μM).^41^ The mechanism of action of AB61 (**7a**) was studied in detail^42^ (Fig. 3). AB61 (**7a**) is efficiently phosphorylated in cancer cell lines but not in fibroblasts which is the reason it shows selectivity toward cancer cells. The resulting ribonucleoside triphosphate (NTP) is incorporated both into cellular RNA and DNA where it causes block of translation and DNA damage, respectively. Interestingly, although tubercidin triphosphate was incorporated into RNA under the same experimental conditions, subsequent RNA translation proceeded smoothly. AB61 trisphosphate is a substrate for mitochondrial DNA polymerase γ and therefore AB61 (**7a**) might interfere with mtDNA replication and mitochondrial functions similarly as described for other nucleoside analogs.^43^ The in vivo studies of xenograft models confirmed the promising properties of AB61 (**7a**) for its further development as an anticancer agent.

**Figure 4:**
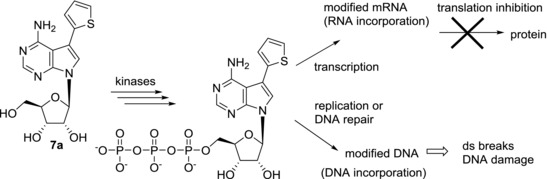
Scheme of the mechanism of action of AB61 (**7a**)

In order to further expand the series of 7‐hetaryl 7‐deazapurine ribonucleosides, a series of their C6 and C2 modified analogues was prepared^44^ (Fig. 3). Within the series, C6‐substituted 7‐(2‐furyl) derivatives (**9a–e**) showed the strongest cytotoxic effect (submicromolar), especially the derivatives with 6‐methoxy (**9a**), 6‐methylsulfanyl (**9b**), and 6‐methyl (**9e**) substituent. On the other hand, introduction of methyl (**10a**) or amino group (**10b**‐**c**) into position 2 resulted in complete loss of activity. Also, 7‐hetaryl‐7‐deazainosines and 7‐deazaguanosines were inactive.^44^ 7‐Ethynyl‐7‐deazaadenosine (**11a**) can be considered as a structural analogue of toyocamycin (**4**). Its cytotoxic effect against cancer cell lines (nanomolar) is even more powerful than that of clinically used cytostatics doxorubicin and clofarabine or tubercidin (**3**).^12^ Substitution of 6‐amino group in 7‐ethynyl‐7‐deazaadenosine (**11a**) generally reduced its cytotoxic activity but 6‐methylsulfanyl (**11b**), 6‐dimethylamino (**11c**), and 6‐methyl (**11d**) derivatives still kept submicromolar cytotoxicity toward some cancer cell lines.^44^ Interestingly, 7‐ethynyl‐6‐methylsulfanyl‐7‐deazapurine ribonucleoside (**11b**) showed only mild cytotoxicity against normal fibroblasts while 6‐dimethylamino (**11c**) and 6‐methyl (**11d**) derivatives were poorly selective. 7‐Alkynyl‐7‐deazaadenosines (**12a**‐**b**) were similarly potent in vitro cytostatics as tubercidin (**3**) but their mechanisms of action are probably different. While inhibition of protein kinase A is considered as one of the cell growth inhibition mechanisms, e.g. of toyocamycin (**4**),^32^ derivative **12a** is a protein kinase A activator.^45^

**Figure 5:**
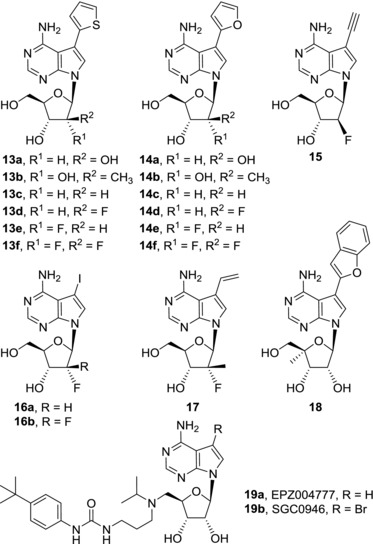
Sugar‐modified derivatives of 7‐substituted 7‐deazaadenosines

Introduction of sugar modifications in 2′‐position of the ribose moiety in 7‐hetaryl‐7‐deazaadenosines (**13a**‐**f**, **14a**‐**f**) led to a decrease in cytotoxic activities^46^, ^47^ (Fig. 5). Still some of the arabinonucleosides (**13a** and **14a**), 2′‐*C*‐methylribonucleosides (**13d**),^46^ 2′‐deoxy‐2′‐fluoroarabinonucleosides (**13d**), and 2′‐deoxy‐2′,2′‐difluororibonucleosides (**13f** and **14f**)^47^ possessed micromolar cytotoxicity. Again it seems that the mechanisms of action between parent ribonucleosides such as AB61 (**7a**) and their sugar‐modified analogues could be different. For instance, the triphosphate derived from 2′‐deoxy‐2′,2′‐difluororibonucleoside **13f** was shown to be an inhibitor of human DNA polymerase α while AB61‐triphosphate was not an inhibitor of the enzyme.^47^ In contrary to 7‐hetaryl‐7‐deazaadenosines, a less profound decrease of activity was observed when cytotoxic activities of 7‐ethynyl‐7‐deazaadenosines (**11a**) and its 2′‐deoxy‐2′‐fluoro‐*arabino*‐analogue **15** were compared. Derivative **15** possessed submicromolar to nanomolar activities against cancer cell lines in vitro, however lower than those of the parent ribonucleoside **11a**.^46^ Submicromolar cytotoxicities were observed among 2′‐fluorinated analogues of 5‐iodotubercidin **16a** and **16b** 47 and 7‐vinyl‐7‐deazaadenine 2′‐fluoronucleoside **17** also showed moderate (micromolar) cytotoxic activity.^48^ Introduction of 4′‐*C*‐methyl group into 7‐hetaryl‐7‐deazaadenosines led to complete loss of cytotoxic effect with the only exception of 2‐benzofuryl derivative **18** with modest (micromolar) activity.^49^ In conclusion, likewise among naturally occurring cytotoxic 7‐deazapurine nucleosides, modifications of the ribose moiety in 7‐substituted 7‐deazapurine nucleosides mostly result in significant decrease of cytotoxic activities.

Structure‐based drug design was used in development of inhibitors of DOT1L methyltransferase. This enzyme is a protein methyltransferase that methylates histone H3 on lysine 79 (H3K79). Aberrant methylation of H3K79 by DOT1L is essential for development of MLL‐rearranged mixed lineage leukemia. The structure of EPZ004777 (**19a**), a selective subnanomolar inhibitor of DOT1L (*K_i_* = 0.3 nM), was derived from structure of *S*‐adenosylhomocysteine (SAH), a cofactor by‐product of methylation catalyzed by DOT1L. Binding of EPZ004777 (**19a**) into SAH‐binding pocket of DOT1L was intensely studied. The residence time of EPZ004777 (**19a**) is much longer (τ ≈ 1 h) compared with SAH (τ = 10 s).^50^ EPZ004777 (**19a**) is capable of selective inhibition of proliferation of MLL‐rearranged cell lines and showed efficacy in vivo in a mouse xenograft model.^51^ Introduction of bromine atom into position 7 of 7‐deazapurine moiety led to compound SGC0946 (**19b**) which showed improved DOT1L inhibitory and MLL‐rearrangement‐related cytotoxic effect as compared with EPZ004777 (**19a**).^52^

**Figure 6:**
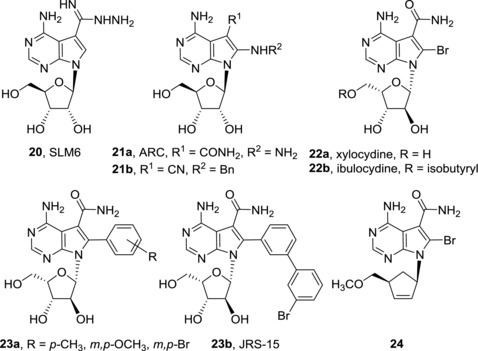
Structures of sangivamycin‐like molecules

In so‐called sangivamycin‐like molecules (SLMs), the carbamoyl group of sangivamycin (**5**) is replaced by other carboxylic‐group‐related substituents and/or additional substituents are attached to position 8 of the 7‐deazapurine moiety (Fig. 6). SLMs, including SLM6 (**20**) and ARC (**21a**), showed potent in vitro cytotoxicity against multiple myeloma cell lines.^53^ The efficacy of SLM6 (**20**) was also confirmed in vivo. Its cytotoxic effect is caused by inhibition of cyclin dependent kinase 9 (CDK9). ARC (**21a**), 8‐hydrazinosangivamycin, attracted attention for its promising cytotoxic activities against colorectal cancer,^54^ melanoma,^55^ and neuroblastoma cells.^56^ Synergistic effect was observed in treatment of cancer cell lines with ARC (**21a**) and a pan‐Bcl‐2 inhibitor ABT‐737 (for structure, see 57).^58^ ARC (**21a**) is a general transcriptional inhibitor acting through inhibition of positive transcription elongation factor b (P‐TEFb), a complex of CDK9/cyclin T1.^59^ ARC (**21a**) induces p53‐independent apoptosis in malignant cells but not in normal cells. It inhibits Akt signaling pathway^56^ and decreases expression of antiapoptotic proteins such as Mcl‐1.^54^, ^55^ Nevertheless, ARC (**21a**) was found inactive in xenograft models in vivo, presumably due to rapid serum clearance.^60^ It was shown that the activity and mechanistic aspects of ARC (**21a**) are identical to those of sangivamycin (**5**) so that the lack of efficacy of ARC (**21a**) is in accord with previous failures of sangivamycin (**5**) in clinical trials.^60^ Compound **21b**, an 8‐substituted toyocamycin analogue, was found to bind HSP70 more efficiently than toyocamycin (**4**) and sangivamycin (**5**) (*K_D_* = 2.8 μM). As inhibition of HSP70 in cancer cell lines induces apoptosis,^61^ derivative **21b** has a potential to possess cytotoxic activity but the data are not yet reported. Xylocydine (**22a**), also called BMK‐Y101, a sugar‐modified analogue of 8‐bromosangivamycin, is a selective inhibitor of cyclin dependent kinases (CDK1, CDK2, CDK7, and CDK9)^62^, ^63^ which demonstrated its antitumor potential in hepatocellular carcinoma both in vitro and in vivo, while being inactive against cervical, prostate, and hepatic carcinoma or lung adenocarcinoma.^64^ Further studies with leukemic HL‐60 cell line showed that xylocydine (**22a**) induces apoptosis in these cells by CDK1 and CDK4 inhibition and upregulation of protein p16^INK4a^, which is a CDK inhibitor.^65^ Isobutyryl ester prodrug of xylocydine, ibulocydine (**22b**), also causes inhibition of CDK7 and CDK9. Subsequent inhibition of RNA polymerase II phosphorylation leads to rapid down‐regulation anti‐apoptotic of proteins Mcl‐1, survivin, and XIAP, thus inducing apoptosis in hepatocellular carcinoma cell lines.^66^ Hepatocellular carcinoma cell lines can be sensitized to TRAIL (tumor necrosis factor‐related apoptosis‐induced ligand)‐induced apoptosis by treatment with ibulocydine (**22b**)^67^ that is also capable of inhibiting growth of hepatocellular carcinoma in a mouse xenograft model.^66^ Combination of radiotherapy and ibulocydine (**22b**) treatment showed promising results both in vitro (apoptotic cell death accompanied with activation of caspases, decrease in Bcl‐2/Bax expression, loss of mitochondrial membrane potential, and release of cytochrome c into cytosol) and in vivo (reduced tumor volume in lung cancer xenografts in mice).^68^ Replacement of an 8‐bromine atom of xylocydine (**22a**) by *p‐*tolyl, *m‐* or *p‐*methoxyphenyl, and *m‐* or *p‐*bromophenyl groups in compounds **23a** led to loss of CDK1 and CDK2 inhibitory activity^69^ and also JRS‐15 (**23b**) with 8‐biphenylyl group is devoid of any CDK inhibiton.^64^ However, JRS‐15 (**23b**) showed cytotoxicity in broader panel of cancer cell lines compared to xylocydine (**22a**), even though these are only moderate (micromolar).^64^ Carbocyclic derivative **24** that shares 7‐deazapurine substitution pattern of xylocydine (**22a**) possesses mild cytotoxic activity against ovarian cancer cell line PA‐1.^70^ It is also important to mention that 8‐substituted 7‐deazapurine are likely to adopt *syn*‐conformation^69^ and binding modes to their molecular target may substantially differ from those of 8‐unsubstituted 7‐deazapurine nucleosides where *anti*‐conformation is preferred. In conclusion, nucleobase modifications of 7‐deazapurine moiety in positions 7 and 8 cannot only dramatically change the cytotoxic activities but it also affects mechanisms of action of particular nucleosides. Mechanisms of action of cytotoxic 7‐deazapurine nucleosides are typically quite complex and often include activation in cells to obtain corresponding nucleotides that can interfere e.g. with DNA and RNA synthesis. It is common that one compound targets more pathways in parallel, which makes it difficult to decide which of them is the most significant. Some 7‐deazapurine nucleosides target specific enzymes such as adenosine kinase, CDKs or protein kinase A and C. It is difficult to predict the mechanism of action of novel analogues of 7‐ and 8‐substituted 7‐deazapurine nucleosides because many compounds from this group significantly differ in their modes of action although their structures are very similar. Even though target‐based design was successfully applied in the development of DOT1L inhibitors, most of discoveries in development of cytotoxic 7‐deazapurine nucleosides are based on serendipity. More focused studies of binding of cytotoxic nucleotides to cellular DNA and RNA polymerases are required in the future in order to move structure‐activity studies into more target‐related research.

### 2.3 Synthetic nucleosides with C6 substituents

**Figure 7:**
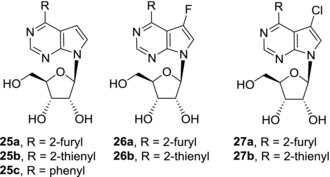
Structures of cytotoxic 6‐(het)aryl‐7‐deazapurine ribonucleosides

Substitution of 6‐amino group of 7‐deazapurine nucleosides by carbon substituents brings further possibilities for structure‐activity relationship studies among 7‐deazapurine nucleosides. As the substitution at position 6 of 7‐deazapurine nucleosides affects, or in some cases even prevents, formation of stable Watson‐Crick base pairs, their mechanisms of action may differ from those of 7‐deazaadenine nucleosides. In our lab, we prepared a large series of 6‐(het)aryl‐7‐deazapurine ribonucleosides **25** and found that they show different cytotoxic activities based on the nature of the substituent (Fig. 7). Derivatives substituted with a small five‐membered heterocycles, e.g. furan (**25a**) or thiophene (**25b**), are nanomolar cytostatics against a broad range of cancer cell lines, while phenyl derivative **25c** is mostly inactive.^71^ In this case, substitution of the phenyl ring did not bring substantial improvement of activity. Further substitution by fluorine in position 7 in derivatives **26a‐b** did not affect the efficacy of the parent compounds, on the other hand 7‐chloroderivatives **27a‐b** were significantly less active or inactive. The decrease of cytotoxic potency in 7‐chloroderivatives is in agreement with the fact that intracellular phosphorylation of **27a** is much less efficient than that of unsubstituted derivative **25a**, indicating intracellular phosphorylation may be limiting the activity. The mechanism of action of 6‐hetaryl‐7‐deazapurine ribonucleosides has not been fully examined yet, however, rapid and powerful cellular RNA synthesis inhibition was observed after treatment with **25a‐b** and **26a**.

**Figure 8:**
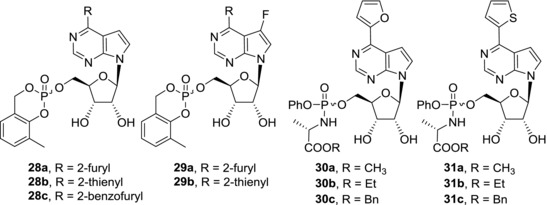
Phosphate‐prodrugs of 6‐(het)aryl‐7‐deazapurine ribonucleosides

Since the efficient phosphorylation seemed to be crucial for cytotoxic activities of 6‐hetaryl‐7‐deazapurine ribonucleosides **25** and **26**, phosphate prodrugs of these compounds were synthesized in efforts to further improve their cytotoxicity (Fig. 8). *Cyclo*Sal pronucleotides of 6‐hetaryl‐7‐deazapurine (**28**) and 6‐hetaryl‐7‐fluoro‐7‐deazapurine (**29**) nucleosides however showed similar or slightly lower cytotoxic activities compared to parent nucleosides in most cancer cell lines.^72^ Again, derivatives with small furyl and thienyl substituents **28a**‐**b** and **29a**‐**b** were more potent than derivatives with bulky substituents such as benzofuryl **28c** which were devoid of any cytotoxic effect. ProTide approach was also applied and phosphoramidate prodrugs based on 6‐hetaryl‐7‐deazapurine nucleosides were prepared.^73^ Nevertheless, nanomolar cytotoxic activities of the parent nucleosides dropped to micromolar in their ProTides, no matter if methyl (**30a**, **31a**), ethyl (**30b**, **31b**), or benzyl (**30c**, **31c**) alanine esters were used. The decrease of activities was presumably caused by efflux of ProTides out of the cells.

**Figure 9:**
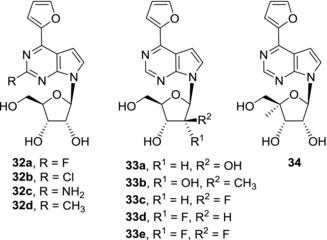
2‐Modified and sugar‐modified derivatives of 6‐hetaryl‐7‐deazapurine nucleosides

Further nucleobase modifications of 6‐hetaryl‐7‐deazapurine nucleosides comprised of introduction of substituents into position 2^74^ (Fig. 9). However, cytotoxicity screening showed significant drop of activities among 2‐substitued derivatives compared to their parent nucleosides. Only some modest (micromolar) activity of 2‐fluoro derivative **32a** was observed, more bulky 2‐chloro‐ (**32b**), 2‐amino‐ (**32c**), and 2‐methyl (**32d**) derivatives were inactive. Also, the ribonucleoside moiety was shown to be crucial for keeping the potency of 6‐hetaryl‐7‐deazapurine nucleosides as all of the sugar‐modified derivatives, such as **33a**‐**33e** and **34**, were devoid of any cytotoxic activity.^49^, ^75^, ^76^ (Fig. 9). Structure‐activity studies of 6‐hetaryl‐7‐deazapurine nucleosides mentioned in this section and those of 7‐hetaryl‐7‐deazapurine nucleosides (Section 2.2) therefore are in agreement with the hypothesis that the modes of action of these classes of compounds are different.

**Figure 10:**
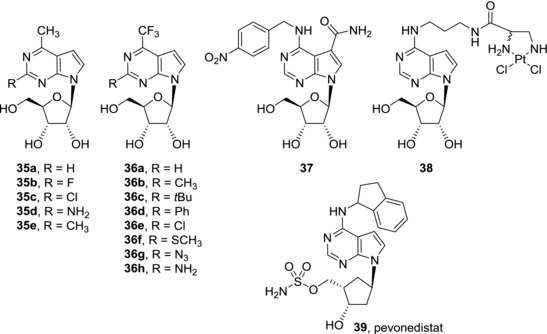
Other examples of cytotoxic 7‐deazapurine ribonucleosides

Replacement of the amino group of tubercidin (**3**) by a methyl group led to 6‐methyl‐7‐deazapurine ribonucleoside (**35a**) (Fig. 10). Compound **35a** is a potent cytotoxic agent with low submicromolar activities against a broad range of cancer cell lines.^44^ Unfortunately, 6‐methyl‐7‐deazapurine ribonucleoside (**35a**) is poorly selective and showed similar cytotoxic effect also against normal fibroblasts. Substitution of compound **35a** in position 2 is not tolerated as the resulting 2‐fluoro (**35b**), 2‐chloro‐ (**35c**), 2‐amino (**35d**), and 2‐methyl (**35e**) derivatives are completely inactive.^74^ 6‐Trifluoromethyl‐7‐deazapurine ribonucleoside (**36a**) possesses similar cytotoxic activities against cancer cell lines as the 6‐methyl analogue **35a**.^77^ Also in this case no 2‐modified derivatives **36b**‐**h** showed any cytotoxicity.^77^

Another option for modification in position 6 is N6‐alkylation of 7‐deazaadenine (Fig. 10). N6‐benzyl and N6‐nitrobenzyl derivatives of tubercidin (**3**), toyocamycin (**4**), and sangivamycin (**5**) were studied as inhibitors of nucleoside transporter 1 (hENT1) which is a protein that plays key role in nucleoside drug uptake. Among the compounds synthesized in the study, N6‐nitrobenzyl derivative of sangivamycin **37** showed both efficient (nanomolar) inhibition of the hENT1 transporter and mild (micromolar) cytotoxicity against several cancer cell lines.^78^ Conjugation of tubercidin (**3**) and cisplatin, a clinically used cytostatic, through N6‐nitrogen of tubercidin furnished derivative **38**. Although compound **38** was able to react with purine residues in a similar way as cisplatin, its cytotoxic activity against cancer cell lines was only weak (micromolar) and dropped by one order of magnitude compared to cisplatin.^79^ Based on these two examples, it could seem that attachment of larger substituents to the N6‐amino group is not suitable for improvement of cytotoxic effect of 7‐deazapurine nucleosides but such conclusion would be too simplistic. Pevonedistat (**39**, MLN4924) is N6‐substituted carbocyclic 7‐deazapurine nucleoside analogue which shows mode of action distinct from those of other 7‐deazapurine nucleosides. Pevonedistat (**39**) is a potent and selective inhibitor of NEDD8‐activating enzyme (NAE) (IC_50_ = 4 nM) that regulates proteasome‐mediated protein degradation.^80^ Accumulation of specific proteins caused by inhibition of NAE leads to deregulation of S‐phase DNA synthesis and subsequently to apoptosis. Despite modest clinical activity of pevonedistat (**39**) in phase I study of patients with acute myeloid leukemia and myelodysplastic syndromes,^81^ it showed significant therapeutic effect in phase I trials against refractory multiple myeloma,^82^ metastatic melanoma,^83^ and advanced solid tumors.^84^ In conclusion, some of 6‐modified‐7‐deazapurine nucleosides show potent cytotoxic effect against cancer cell lines. With the exception of pevonedistat (**39**), their mode of action is not well understood yet and most of the reports focused only on structure‐activity studies. Nevertheless, it seems that the mechanisms of action of 6‐substituted‐7‐deazapurine nucleosides vary within the group and, at the same time, they are significantly different from those of 7‐deazaadenine nucleosides.

### 2.4 Fused nucleosides

**Figure 11:**
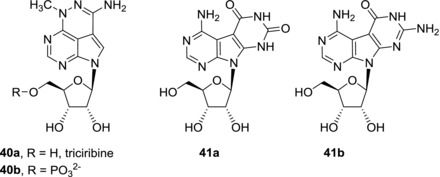
Structures of triciribine and Janus‐type tricyclic nucleosides

7‐Deazapurine nucleobase can be further extended by annulation with other aromatic rings leading to fused (tricyclic) nucleosides (Fig. 11). Triciribine (**40a**), also known as TCN or API‐2, was synthesized already in 1971.^85^ Triciribine (**40a**) suffers from poor solubility and therefore its soluble 5′‐monophosphate prodrug **40b** (TCN‐P) is often used. Antineoplastic activities of triciribine (**40a**) and TCN‐P (**40b**) both in vitro and in vivo were studied in 1980s and early 1990s and were reviewed previously.^86^ Clinical trials were halted due to toxicity in high dosing of TCN‐P (**40b**) and mild efficacy presumably due to its low bioavailability. However, recently it was shown that the bioavailability can be significantly improved by application of phosphoramidate prodrug approach.^87^ Triciribine (**40a**) acts through selective inhibition of Akt kinase^88^ and therefore is effective in Akt‐overexpressing tumors such as pancreatic and ovarian. Recently, synergistic effects were observed in combinations of TCN‐P (**40b**) with other antineoplastic compounds such as gemcitabine^89^ and tipifarnib.^90^ Clinical studies of other combination therapies with TCN‐P (**40b**) are currently under way.

Pyrimido‐fused nucleosides are also called dual bases or Janus‐type tricyclic nucleosides because they can present Watson‐Crick donor/acceptor array of one nucleobase on one face and different donor/acceptor array on the other face and therefore can form stable base pairs with two different complementary nucleobases. The Janus‐type nucleobases such as **41a‐b** showed only moderate (micromolar) cytotoxic activities and later on they became conceived more as antivirals.^91^, ^92^

**Figure 12:**
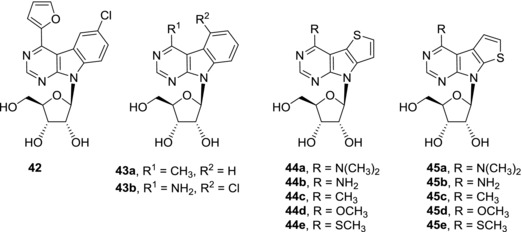
Structures of cytotoxic benzo‐ and thieno‐fused 7‐deazapurine nucleosides

Most of the benzo‐fused 7‐deazapurine nucleosides prepared so far lack any cytotoxic activity against cancer cell lines (Fig. 12). Derivatives **42** and **43a**‐**b** are the only examples of weakly active (micromolar) compounds.^93^, ^94^ On the other hand, thieno‐fused nucleosides, which are isosteric to above‐mentioned benzo‐fused nucleosides, showed interesting cytotoxic effects^95^ (Fig. 12). Substituents in position 6 of 7‐deazapurine part of the nucleobase play an important role in structure‐activity studies. While dimethylamino derivatives (**44a**, **45a**) were inactive and amino derivatives (**44b**, **45b**) possess only moderate (micromolar) cytotoxic activities, methyl (**44c**, **45c**), methoxy (**44d**, **45d**), and methylsulfanyl (**44e**, **45e**) derivatives are potent cytotoxic compounds with submicromolar activities against cancer cell lines of different origin. The position of sulfur heteroatom has an impact on selectivity of the compounds. Series of compounds with thieno[2′,3′:4,5]pyrrolo[4,5‐*d*]pyrimidine nucleobase **44** was similarly toxic towards both cancer cells and normal fibroblasts but series of compounds with thieno[3′,2′:4,5]pyrrolo[4,5‐*d*]pyrimidine nucleobase **45** showed selectivity towards cancer cells. Initial studies of mode of action of compounds **44c**‐**e** and **45c**‐**e** revealed RNA synthesis inhibition and decrease in mitotic cell fraction in cell cycle analysis. Both compounds **44e** and **45e** are efficiently phosphorylated both in normal and cancer cells indicating that cell‐type specific phosphorylation is not a reason for the different selectivities of series **44** and **45**.^95^ In conclusion, even though the family of fused nucleosides is relatively small, it has already brought interesting compound hits and deserves further development as a source of cytotoxic nucleosides. Detailed study of mechanism of action of these compounds is particularly needed and is under way.

## 3 ADENOSINE KINASE INHIBITORS

### 3.1 Inhibitors of mammalian adenosine kinases

**Figure 13:**
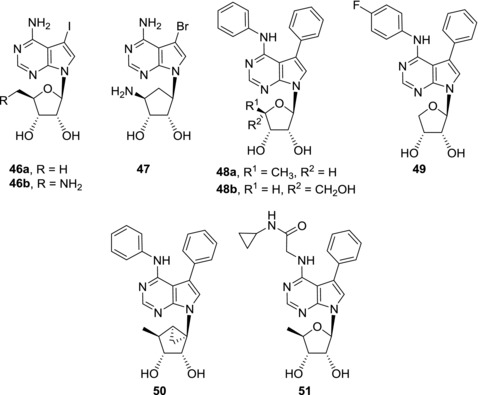
Examples of 7‐deazapurine nucleosides as inhibitors of mammalian ADKs

Adenosine kinase (ADK) is an enzyme that catalyzes the conversion of adenosine to adenosine‐5′‐*O*‐monophosphate (AMP). Extracellular adenosine is a ligand of adenosine receptors which regulates heart rate, neurotransmitter release in brain, and inflammatory response. Stimulation of adenosine receptors is connected with anticonvulsant, analgesic, and anti‐inflammatory activity. Inhibition of ADK leads to increased adenosine levels and so ADK inhibitors possess similar effect as adenosine receptor agonists. Moreover it was shown that ADK inhibitors show less side effects in anti‐seizure activity assays and therefore can be considered as a promising class of anticonvulsants.^96^ Data from Section 3 is summarized in Table 2. 5′‐Deoxy‐5‐iodotubercidin (**46a**) is a naturally occurring nucleoside isolated from a marine red alga (Fig. 13). 5′‐Deoxy‐5‐iodotubercidin (**46a**) is a potent inhibitor of mammalian adenosine kinases^37^, ^97^ (IC_50_ = 9 nM against human ADK) and does not bind to adenosine receptors.^98^ Also other 7‐halogenated 7‐deazaadenine nucleosides show ADK inhibitory activity. Although 5‐iodotubercidin (**6a**)^37^, ^97^ and 5‐bromotubercidin (**6b**)^22^, ^97^ are less potent ADK inhibitors than 5′‐deoxy‐5‐iodotubercidin (**46a**), they still possess submicromolar IC_50_ values. Both 5′‐deoxy‐5‐iodotubercidin (**46a**) and 5‐iodotubercidin (**6a**) showed anti‐seizure activities in vivo,^96^, ^97^ however, these compounds are not suitable for clinical use due to behavioral effects (decreased locomotor activity, hypothermia, and muscle flaccidity)^98^ and cytotoxicity,^39^ respectively. In order to obtain more suitable clinical candidates, a large structure‐activity study was performed.^97^ It was shown that the presence of a halogen atom in position 7 is crucial for the ADK inhibitory activity. The most potent activities were observed in derivatives with amino, chloro, or methylsulfanyl group in position 6 and 5′‐amino group. For instance, derivative **46b** showed subnanomolar human ADK inhibition (IC_50_ = 0.6 nM). Compound **46b** showed anti‐seizure activity in an in vivo model. However, it seems that anti‐seizure activities generally do not fully correlate with ADK inhibitory effects, presumably due to different pharmacological properties of the tested compouds.^97^

**Table 2:** ADK inhibitory activity of 7‐deazapurine nucleosides

Compound	IC_50_ [μM] (human ADK)	IC_50_ [μM] (*Mtb*‐ADK)	Ref.
7‐Iodotubercidin (**6a**)	0.026	nd^a^	97
7‐Bromotubercidin (**6b**)	0.12	nd	97
**7c**	>5	>10	100
**11a**	0.20	0.33	100
**35a**	>10	8.8	107
**35b**	>10	>10	74
**35c**	>10	>5	74
**35d**	>10	>10	74
**35e**	>20	>5	74
**46a**	0.009	nd	97
**46b**	0.0006	nd	97
**47**	0.00047	nd	101
**48a**	0.0005	nd	101
**48b**	0.00047	nd	102
**49**	0.006	nd	103
**50**	0.088	nd	103
**51**	0.003	nd	105
**52**	>10	0.6	100
**53a**	>20	0.023	107
**53b**	>20	0.028	107
**54a**	0.3	0.0075	107
**54b**	2.1	0.0145	107
**55**	>10	0.0012	74

a nd, not determined

ADK inhibitors are also promising analgetics. Carbocyclic 7‐bromo‐7‐deazapurine nucleoside **47** is a subnanomolar inhibitor of ADK (IC_50_ = 0.47 nM against human ADK) which showed antinociceptive activity in animal acute, inflammatory, and neuropathic pain models (Fig. 13).^99^

Replacement of iodine atom in 5‐iodotubercidin (**6a**) by ethynyl group led to drop of ADK inhibitory activity, however, resulting 7‐ethynyl‐7‐deazaadenosine (**11a**) still showed submicromolar ADK inhibition.^100^ On the other hand, tubercidin derivatives with larger hydrophobic groups in position 7, such as 7‐phenyl‐7‐deazaadenosine (**7c**), do not inhibit human ADK.^100^ Despite this fact, when 7‐phenyl group is combined with phenyl group attached to N6 nitrogen atom, the potent ADK inhibition in these so called diaryl derivatives is restored^101^ (Fig. 13). Further sugar modifications led to 5′‐deoxy derivative **48a** 101 and α‐l‐lyxofuranosyl derivative **48b** 102 that are subnanomolar human ADK inhibitors (IC_50_ = 0.5 and 0.47 nM, respectively). Compound **48a** appeared to be a potent anticonvulsant in a rat seizure model,^101^ while compound **48b** showed moderate anti‐inflammatory effect both in rat carrageenan paw edema model and rat skin lesion model.^102^ Also erythrofuranosyl diaryl‐derivative **49** is a nanomolar ADK inhibitor (IC_50_ = 6 nM).^103^ Derivative **49** showed analgesic activity in several animal pain models. The effect of compound **49** in rat formalin paw pain model was similar to that of standard opioid analgesic morphine.^103^ Recently, submicromolar ADK inhibition was also observed in carbocyclic diaryl nucleosides such as compound **50** (IC_50_ = 0.088 μM).^104^ However, in vivo activities of this class of compounds have not been studied yet. Despite potent activities of diaryl nucleosides, the compounds generally suffer from poor water solubility. Replacement of N6‐phenyl group by glycinamide substituent led to compound **51** which showed improved solubility and nanomolar ADK inhibitory effect at the same time (IC_50_ = 3 nM).^105^ In vivo animal pain models provided promising results as derivative **51** was shown to be more potent than morphine.^105^ Unfortunately, prolonged administration of compound **51** is connected with lethal toxicity and therefore further development of this compound was discontinued. In spite of this fact human ADK inhibitors still represent a very promising group of compounds and deserve attention for their antinociceptive, anti‐seizure and anti‐inflammatory activities.

### 3.2 Inhibitors of mycobacterial adenosine kinase

**Figure 14:**
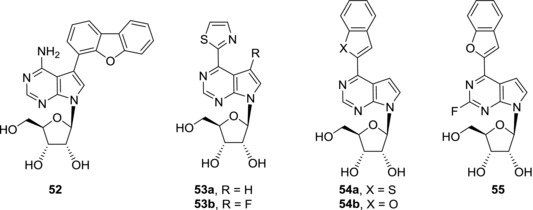
Examples of 7‐deazapurine nucleosides as inhibitors of *Mtb*‐ADK

Many pathogens lack enzymes for de novo purine synthesis and use purine salvage pathway instead. In this pathway purine nucleotides are formed from nucleobases and phosphoribosyl pyrophosphate or purine nucleosides are phosphorylated by kinases, e.g. adenosine kinase. Targeting ADK of the pathogen can therefore lead to anti‐microbial compounds. *Mycobacterium tuberculosis* expresses enzymes from both salvage pathway and de novo purine synthesis pathway; however, the interdependence and regulation of these processes remain unclear.^106^ For instance, 7‐ethynyl‐7‐deazaadenosine (**11a**) is a submicromolar inhibitor of ADK of *Mycobacterium tuberculosis* (*Mtb‐*ADK) (IC_50_ = 0.33 μM) and showed antimycobacterial activities both against *Mycobacterium bovis* and *Mycobacterium tuberculosis*.^100^ Nevertheless, poor ADK inhibition selectivity and cytotoxicity of **11a** are not desirable. On the other hand, 7‐hetaryl‐7‐deazaadenosines with bulky substituents have been already mentioned as compounds that are non‐cytotoxic and do not inhibit human ADK.^12^, ^100^ For instance, dibenzofuryl derivative **52** is a submicromolar inhibitor of *Mtb*‐ADK (IC_50_ = 0.6 μM) that is devoid of any cytotoxicity towards human fibroblasts and possesses micromolar antimicrobial activity against *Mtb* strains^100^ (Fig. 14). Also 6‐hetaryl‐7‐deazapurine ribonucleosides were shown to selectively inhibit with *Mtb*‐ADK in submicromolar concentrations.^107^ Derivatives with small hetaryl groups such as **53a** and **53b** (IC_50_ = 23 and 28 nM, respectively) are highly selective to *Mtb*‐ADK with selectivity index over 10,000. However, these compounds are cytotoxic and their antimycobacterial activities against *Mycobacterium bovis* are poor. On the other hand, derivatives with more bulky substituents such as benzothienyl (**54a**) and benzofuryl (**54b**) are very powerful *Mtb*‐ADK inhibitors (IC_50_ = 7.5 and 14.5 nM, respectively), they show good selectivity and are non‐cytotoxic.^107^ Nevertheless, these compounds lack antimycobacterial activity against *Mycobacterium bovis*. The reason for poor correlation between *Mtb*‐ADK inhibition and antimycobacterial activity could be poor penetration of the tested compounds through the mycobacterial cell wall or bypassing *Mtb*‐ADK inhibition through alternative purine synthesis pathways. The presence of a fluorine atom in 6‐hetaryl‐7‐deazapurine ribonucleosides can further improve both *Mtb*‐ADK inhibitory activity and selectivity. Benzofuryl derivative (**55**) is a nanomolar inhibitor of *Mtb*‐ADK (IC_50_ = 1.2 nM) but again derivative **55** showed only modest activity against *Mtb*.^74^ On the other hand, 6‐methyl‐7‐deazapurine ribonucleoside (**35a**) is a weak *Mtb*‐ADK inhibitor (IC_50_ = 8.8 μM) but showed strong submicromolar activity against *Mycobacterium bovis*.^107^ In this case, the mode of antimycobacterial activity is presumably independent of *Mtb*‐ADK and rather suggests a more general cytotoxic mechanism as compound **35a** is highly cytotoxic also toward human cells. This is in agreement with the fact that its 2‐substituted analogues **35b**–**e** showed neither antimycobacterial activity nor cytotoxicity.^74^ In conclusion, the most potent 7‐deazapurine nucleoside inhibitors of *Mtb*‐ADK are only weakly active in antimycobacterial assays. Poor cellular uptake could be one of the reasons for this fact, however, initial attempts to improve the cell penetration by lipophilic prodrugs failed.^107^ Therefore, showing that standard prodrug approaches for penetration for eukaryotic cells may not be effective in mycobacteria and requires further studies.

## 4 NUCLEOSIDES WITH ANTIVIRAL ACTIVITIES

### 4.1 Nucleosides with anti‐HCV activities

#### 4.1.1 Ribonucleosides

**Figure 15:**
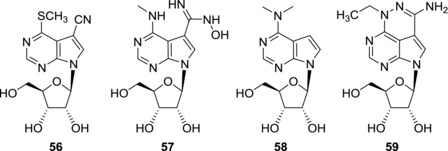
Examples of anti‐HCV 7‐deazapurine ribonucleosides

Hepatitis C virus (HCV) is an RNA virus of the family *Flaviviridae*. It uses RNA‐dependent RNA polymerase (NS5B) for replication of its genetic information. Because human cells do not express any RNA‐dependent RNA polymerase and HCV RNA polymerase (NS5B) is structurally different from human RNA and DNA polymerases, the viral enzyme became a target for new anti‐HCV compounds development. Thanks to their close resemblance to natural substrates of the viral RNA polymerases, many 7‐deazapurine nucleosides were studied as potential antiviral candidates not only for HCV but also other RNA viruses that will be mentioned later in the text. Structure‐activity studies of nucleobase‐modified analogues of naturally occurring 7‐deazapurine antibiotics such as tubercidin (**3**) and toyocamycin (**4**) brought several interesting structures with anti‐HCV activities (Fig. 15). Modifications of toyocamycin (**4**) in positions 6 and 7 led to nucleosides **56** and **57** that showed submicromolar activities in HCV replicon assays. EC_50_ and CC_50_ values for compounds mentioned in Section 4.1 are listed in Table 3. Compounds **56** and **57** are non‐cytotoxic and therefore they both represent good lead structures for anti‐HCV drug development.^108^ Also other 6‐ and 7‐modified‐7‐deazapurine ribonucleosides showed interesting in vitro anti‐HCV activities.^44^ 7‐Furyl derivatives **9a** and **9c** showed submicromolar anti‐HCV activities in both HCV 2A and HCV 1B replicon assays. Both **9a** and **9c** were non‐cytotoxic to the HCV replicon cells but **9a** showed submicromolar cytotoxicity towards normal fibroblasts and therefore it cannot be considered as suitable for further development as an anti‐HCV agent. Also 7‐ethynyl derivative **11c** possessed submicromolar anti‐HCV activity and no cytotoxicity in HCV replicon assay but was cytotoxic to fibroblasts.^44^ On the other hand, 6‐*N,N*‐dimethylamino‐7‐deazapurine ribonucleoside (**58**) seems to be more suitable lead structure because it showed submicromolar activities and no cytotoxicity in HCV replicon assay and only weak cytotoxicity towards fibroblasts. Sangivamycin‐like molecule ARC (**21a**) that was developed as an anti‐cancer agent showed in vitro anti‐HCV activity while being non‐toxic to host cells.^109^ It was found out that anti‐HCV activity of ARC (**21a**) is not caused by transcription or translation inhibition but the molecular target of ARC (**21a**) remains unknown. Also another antineoplastic compound, triciribine (**40a**), showed moderate anti‐HCV activity (EC_50_ = 2 μM) and low cytotoxicity.^110^ Triciribine analogue **59** was more potent (EC_50_ = 1 μM) and remained non‐cytotoxic.^110^ Janus‐type nucleosides **41a**‐**b** showed moderate anti‐HCV activities (EC_50_ = 5.7 and 3 μM, respectively) which was accompanied by cytotoxicity toward Vero, CEM, and PBM cell lines.^91^ Anti‐HCV screening of thieno‐fused 7‐deazapurine ribonucleosides showed that derivatives **44c**,**e** and **45c‐e** possess submicromolar activities and do not show any cytotoxic effect against the replicon cells.^95^ The anti‐HCV potency of these compounds is similar to that of anti‐HCV agent mericitabine. However, thieno‐fused 7‐deazapurine nucleosides are mostly cytotoxic to fibroblasts and only derivative **45c** was devoid of this cytotoxic effect. In conclusion, despite interesting anti‐HCV activities of some 7‐deazapurine ribonucleosides, the anti‐HCV effect is often accompanied by cytotoxicity to normal cells and therefore further modifications of the lead structures are necessary in order to obtain suitable anti‐HCV drug candidates.

**Table 3:** Anti‐HCV activities of 7‐deazapurine nucleosides

Compound	EC_50_ [μM] (replicon)^a^	CC_50_ [μM] (replicon)	Ref.
7‐Deazapurine ribonucleosides			
**9a**	0.02 (1B); 0.03 (2A)	>44 (1B); > 44 (2A)	44
**9c**	0.06 (1B); 0.14 (2A)	>44 (1B); > 44 (2A)	44
**11c**	0.07 (1B); 0.10 (2A)	>44 (1B); > 44 (2A)	44
**40a**	2	>300	110
**41a**	5.7	5.7	91
**41b**	3	<10	91
**44c**	0.11 (1B); 0.06 (2A)	>44 (1B); >44 (2A)	95
**44e**	0.47 (1B); 0.34 (2A)	>44 (1B); >44 (2A)	95
**45c**	0.06 (1B); 0.24 (2A)	>44 (1B); >44 (2A)	95
**45d**	0.23 (1B); 0.40 (2A)	>44 (1B); >44 (2A)	95
**45e**	0.13 (1B); 0.80 (2A)	>44 (1B); >44 (2A)	95
**56**	0.4	>250	108
**57**	0.6	>200	108
**58**	0.27 (1B); 0.97 (2A)	>44 (1B); >44 (2A)	44
**59**	1	150	110
Sugar‐modified 7‐deazapurine nucleosides			
**17**	1.8	4.5	48
**60a** (MK‐0608)	0.3	>100	111
**60b**	0.07	>100	116
**60c**	0.13	∼50	116
**60d**	0.24	40	116
**60e**	0.37 (1A); 0.38 (1B)	19.75 (1A); 26.27 (1B)	46
**60f**	0.09	nd	117
**61**	>50	>100	111
**62**	3	>100	118
**63a**	>50	nd^b^	119
**63b**	>50	nd	119
**64**	1.9	nd	120
**65**	>100	nd	120
**66**	1.8	nd	120
**67**	23.8	nd	121
**68**	33.8	nd	121
**69a**	18	>300	110
**69b**	15	>200	110
**70a**	2.66; >10	1165; >10	48, 124
**70b**	1.1	10.4	48
**70c**	24	nd	117
**70d**	35	nd	120
**71**	0.9	>50	125
**72a**	0.11	nd	126

a if replicon is not mentioned, the genotype is not specified in the original article;

b nd, not determined

#### 4.1.2 Sugar‐modified nucleosides

**Figure 16:**
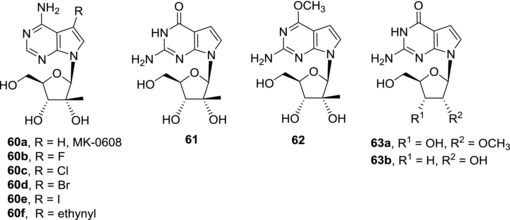
Examples of sugar‐modified anti‐HCV 7‐deazapurine nucleosides

Decreased cytotoxic activities of sugar‐modified 7‐deazapurine nucleosides compared to corresponding ribonucleosides have already been mentioned. Because anti‐HCV activities of 7‐deazapurine ribonucleosides are often accompanied by cytotoxicity, sugar modifications seem to be suitable for restriction of the cytotoxic effect. Among the sugar‐modified nucleosides (such as arabinosides, 3′‐deoxyribonucleosides, and 2′‐*O*‐methylribonucleosides) 2′‐*C*‐methylribonucleosides (more precisely their NTPs) are usually the best HCV RNA polymerase inhibitors^111^ (Fig. 16). 7‐Deaza‐2′‐*C*‐methyladenosine (**60a**),^112^ also known as MK‐0608, was thoroughly studied as an anti‐HCV drug candidate.^111^ It showed submicromolar activity against HCV in a replicon assay and is non‐cytotoxic. MK‐0608 (**60a**) is efficiently phosphorylated in cells and the corresponding NTP is a potent inhibitor of HCV NS5B RNA polymerase. In fact, the NTP is incorporated into the growing RNA chain and acts as a chain terminator.^111^ A single mutation in the NS5B RNA polymerase, S282T, leads to a resistance to MK‐0608 (**60a**).^111^ The anti‐HCV activity of MK‐0608 (**60a**) was confirmed in HCV‐infected chimpanzees where significant reduction of viral load was observed but viral loads rebounded after dosing ended in all tested animals.^113^ Combinational therapy with MK‐0608 (**60a**) and an HCV NS3/4A protease inhibitor vaniprevir (MK‐7009), however, resulted in sustained virological response of viral negativity 6 months after treatment in chimpanzees.^114^ Combinational treatment with MK‐0608 (**60a**) was also successful in HCV‐infected human hepatocyte chimeric mice. The combination of MK‐0608 (**60a**) and another HCV NS3/4A protease inhibitor telaprevir led to sustained virological response but only in high dose regimen or in triple combination therapy with MK‐0608 (**60a**), telaprevir, and interferon.^115^ In spite of these promising results, clinical trials with MK‐0608 (**60a**) have been halted for undisclosed reasons.

Halogen atoms are tolerated in position 7 of 7‐deaza‐2′‐*C*‐methyladenosine. All 7‐halogenated deaza‐2′‐*C*‐methyladenosines **60b**‐**e** showed submicromolar activities in HCV replicon assays.^46^, ^116^ The anti‐HCV activity of the derivatives decreases with the increasing size of the halogen, on the other hand cytotoxicity was found to increase. Therefore only fluoro‐derivative **60b** shows desirable properties and fourfold higher potency compared to 7‐unsubtituted derivative MK‐0608 (**60a**).^116^ The increased potency is presumably caused by improved cellular uptake of **60b**. Introduction of a 7‐ethynyl group in compound **60f** brought significant anti‐HCV activity but unfortunately cytotoxicity of this derivative was not reported. The corresponding nucleoside triphosphate efficiently inhibited HCV RNA polymerase (IC_50_ = 0.75 μM).^117^

Effective cellular permeation and intracellular phosphorylation play a crucial role in development of anti‐HCV agents targeting HCV RNA polymerase. Although 7‐deazaguanine nucleosides (in their triphosphate forms) are potent inhibitors of NS5B RNA polymerase, often more powerful than corresponding 7‐adenine nucleosides,^111^ they perform poorly in the HCV replicon assays. For instance, 7‐deaza‐2′‐*C*‐methylguanosine **61** was shown to be completely inactive.^111^ Even its prodrug, 6‐*O*‐methyl derivative **62** that should be more lipophilic and therefore should enter the cells more easily, is devoid of any anti‐HCV activity.^118^ Also phosphate prodrug approach failed to bring promising anti‐HCV compounds as the prodrugs were poorly active and/or cytotoxic.^118^ Also other sugar‐modified 7‐deazaguanine nucleosides, such as 2′‐*O*‐methylribonucleoside **63a** and 3′‐deoxyribonucleoside **63b** were strong HCV RNA polymerase inhibitors as NTPs^111^, ^119^ but again their phosphate prodrugs were only weakly active or inactive.^119^

**Figure 17:**
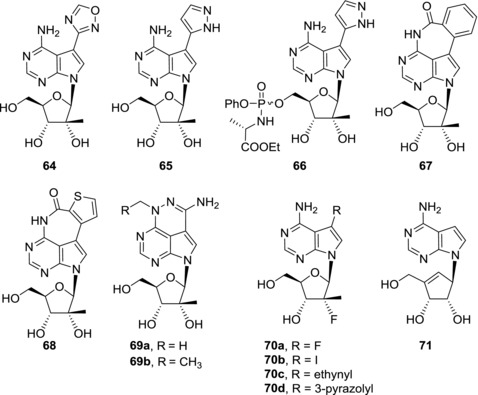
Examples of anti‐HCV derivatives of 7‐(hetaryl)‐7‐deaza‐2′‐*C*‐methyladenosines and 7‐deazaneplanocin A

7‐Hetaryl‐7‐deaza‐2′‐*C*‐methyladenosines generally show modest (micromolar) anti‐HCV activities^46^, ^120^, ^121^ (Fig. 17). The strongest anti‐HCV effect (EC_50_ = 1.9 μM) was observed for oxadiazolyl derivative **64**.^120^ The corresponding NTP is a submicromolar inhibitor of HCV RNA polymerase (IC_50_ = 0.5 μM). Derivative **64** is orally available in rats and high levels of NTP were detected in rat liver. In contrary to 7‐deazaguanine nucleosides, pyrazolyl derivative **65**, which was inactive in HCV replicon assay, was turned to a low micromolar anti‐HCV compound **66** (EC_50_ = 1.8 μM) using ProTide prodrug approach.^120^ Unfortunately, cytotoxicity data were not reported for compounds **64**–**66**.

Tetracyclic 2′‐*C*‐methylribonucleosides **67** and **68** are excellent inhibitors of NS5B RNA polymerase when converted to NTPs (IC_50_ = 0.10 and 0.12 μM, respectively), but their in vitro anti‐HCV activity is only moderate and again, the cytotoxicity data are missing.^121^ Also 2′‐*C*‐methylribonucleoside derivatives of triciribine **69a** and **69b** are only weakly active in HCV replicon assay and significant drop in activities compared to those of corresponding ribonucleosides **40a** and **59** was observed.^110^

Nucleosides with fluorine atoms attached to sugar moiety are also common among compounds with anti‐HCV activity (Fig. 17). Currently, the only approved NS5B polymerase inhibitor, sofosbuvir, is a phosphate prodrug of a 2′‐deoxy‐2′‐fluoro‐2′‐*C*‐methylribonucleoside.^122^, ^123^ Therefore, 7‐Deazapurine 2′‐deoxy‐2′‐fluoro‐2′‐*C*‐methylribonucleosides were also prepared. 7‐Fluoro‐7‐deaza‐2′‐deoxy‐2′‐fluoro‐2′‐*C*‐methyladenosine (**70a**) showed promising activity in HCV replicon assay (EC_50_ = 2.7 μM) and low cytotoxicity^124^ but was found inactive in a different study.^48^ A structure‐activity relationship study of 7‐deazapurine 2′‐deoxy‐2′‐fluoro‐2′‐*C*‐methylribonucleosides identified only 7‐iodo derivative **70b** and 7‐vinyl derivative **17** as compounds that possess moderate anti‐HCV activity, however, derivative **17** was also cytotoxic.^48^ NTPs derived from 7‐ethynyl derivative **70c** 117 and 7‐(pyrazol‐3‐yl) derivative **70d** 120 are both excellent inhibitors of HCV RNA polymerase (IC_50_ = 0.4 and 0.1 μM, respectively) but the nucleosides are inactive or poorly active in cell‐based assay. In contrary to sofosbuvir, in this case again the ProTide prodrug approach failed to improve the anti‐HCV activities of the parent nucleosides.

7‐Deazaneplanocin A (**71**), a carbocyclic nucleoside, showed micromolar activity in HCV replicon assay and was non‐cytotoxic in 100 μM concentration.^125^ The potency of 7‐deazaneplanocin A (**71**) is similar or even better than that of known anti‐HCV compounds 2′‐fluoro‐2′‐deoxy‐2′‐*C*‐methylcytosine and 2′‐*C*‐methylcytosine. 7‐Substituted analogues of 7‐deazaneplanocin A showed either weaker anti‐HCV activity or increased cytotoxicity so that unsubstituted derivative **71** seems to be the most promising lead compound in this series.

**Figure 18:**
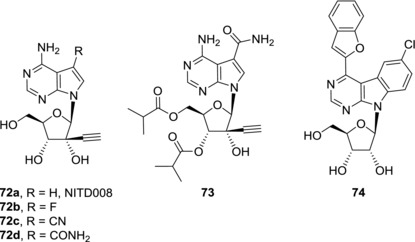
Examples of anti‐DENV 7‐deazapurine nucleosides

7‐Deaza‐2′‐ethynyl‐adenosine (**72a**), also known as NITD008 (Fig. 18), was developed as an anti‐dengue compound.^126^ Because HCV and dengue virus both belong to *Flaviviridae*, NITD008 (**72a**) also possesses anti‐HCV activity.^126^ Despite its anti‐HCV potency, further research related to NITD008 (**72a**) was focused on its anti‐dengue effect only.

Despite the effort made in development of new HCV RNA polymerase inhibitors as anti‐HCV agents, none of the 7‐deazapurine nucleosides proceeded to clinical use. One of the reasons for this fact could be toxicities caused by off target effects as many of other nucleoside NS5B polymerase inhibitors showed to be substrates of human RNA polymerase II and human mitochondrial RNA polymerase.^127^ Finding proper balance between NS5B polymerase inhibition and selectivity, efficient cellular phosphorylation and cell penetration as well as cytotoxicity remains difficult. Still many of the HCV RNA polymerase inhibitors could be useful in development of compounds active against related RNA viruses from *Flaviviridae* family thanks to structural similarities among viral RNA polymerases. Efforts made in this field will be mentioned in the following sections.

### 4.2 Nucleosides with anti‐dengue activities

Dengue fever is a mosquito‐borne infectious disease caused by dengue virus (DENV). DENV is a membrane‐enveloped positive‐strand RNA virus that also belongs to *Flaviviridae*. Currently, there is no specific dengue treatment available. DENV RNA polymerase is one of suitable targets for development of anti‐dengue compounds. Incorporation of unnatural nucleotides into viral RNA could inhibit viral RNA replication and/or hamper function of the viral RNA. Data from Section 4.2 is summarized in Table 4. 6‐Methyl‐7‐deazapurine ribonucleoside (**35a**) showed potent anti‐dengue activity both in a replicon assay (EC_50_ = 0.877 μM) and in an infectivity assay.^128^ Unfortunately, cytotoxicities against host cells were not reported in this study, however, 6‐methyl‐7‐deazapurine ribonucleoside (**35a**) was shown to be highly cytotoxic towards human fibroblasts^107^ and therefore anti‐dengue activity caused by unspecific cytotoxicity of the compound **35a** cannot be ruled out. On the other hand, 7‐deaza‐2′‐*C*‐methyladenosine (**60a**) was able to reduce viral loads in a mouse dengue fever viremia model but its mechanism of action against DENV has not been studied.^129^ Replacement of the 2′‐*C*‐methyl group by ethynyl group led to derivative NITD008 (**72a**). NITD008 (**72a**) showed submicromolar activity against DENV in vitro (EC_50_ = 0.64 μM) and was devoid of cytotoxicity against host cells (Fig. 18).^126^ Further studies showed that NITD008 (**72a**) is phosphorylated in vivo to its NTP^130^ which then acts as a chain inhibitor of RNA‐dependent RNA polymerase of DENV.^126^, ^131^ NITD008 (**72a**) is orally available, reduced viral loads in a mouse model and protected infected animals from death but showed toxicity when treatment was longer than one week.^126^ Compound **72b**, a 7‐fluoro analogue of NITD008, showed slightly increased anti‐dengue activity in vitro compared to NITD008 (**72a**) (EC_50_ = 0.42 μM) but also increased cytotoxicity.^130^ On the other hand, compounds with CN (**72c**) and CONH_2_ (**72d**) substitutions in position 7 showed lower anti‐dengue activities (EC_50_ = 3.1 and 2.0 μM, respectively).^130^ Compound **72d** suffers from poor oral bioavailability in mice and rats.^132^ In order to improve its gastrointestinal absorption and cell penetration, its prodrug, compound **73**, was prepared. Derivative **73** showed both better anti‐dengue activity in vitro (EC_50_ = 0.54 ‐ 0.71 μM, depending on DENV strain) and improved bioavailability than the parent nucleoside **72d**.^132^ Compound **73** efficiently inhibits viral RNA synthesis and was found to be more potent than NITD008 (**72a**) in an in vivo mouse model. Nevertheless, in vivo toxicity in higher doses precluded further development of compound **73** as an anti‐dengue agent. Some benzo‐fused 7‐deazapurine ribonucleosides also showed submicromolar activities against DENV but in case of compounds **42** 94 and **43b** 94 the anti‐dengue effect was presumably caused by unspecific cytotoxicity towards host cells. Only benzofuryl derivative **74** possesses submicromolar anti‐dengue activity in vitro (EC_50_ = 0.976 μM) and is non‐cytotoxic at the same time.^93^ Compound **74** therefore represents a good lead structure for further development of compounds with anti‐dengue effect.

**Table 4:** Anti‐dengue activities of 7‐deazapurine nucleosides

Compound	EC_50_ [μM] (assay)	CC_50_ [μM]	Ref.
**35a**	0.877 (replicon)	nd^a^	128
**42**	0.238 (replicon)	nd	93
**43b**	0.85 (Vero cells)	1.14	95
**72a**	0.64; 0.7 (CFI)	nd; >100	126, 130
**72b**	0.42 (CFI)	44	130
**72c**	3.1 (CFI)	>100	130
**72d**	2.0 (CFI)	62	130
**73**	0.54‐0.71 (CFI)	nd	132
**74**	0.976 (replicon)	nd	93

a nd, not determined

Even though none of the 7‐deazapurine nucleosides reached clinical trials, the preclinical studies showed that inhibitors of viral RNA synthesis are able to gain in vivo antiviral effect and further development of structurally related compounds could lead to clinical candidates.

### 4.3 Nucleosides with antiviral activities against other viruses

**Figure 19:**
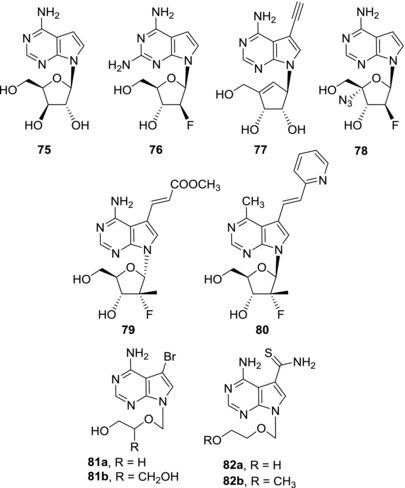
Examples of 7‐deazapurine nucleosides and acyclic nucleosides active against other viruses

7‐Deazapurine nucleosides were subjected to antiviral activity screening against other viruses than HCV and DENV. The largest structure‐activity relationship studies against both DNA and RNA viruses were mostly performed in the 1980s.^31^, ^35^, ^133^, ^134^, ^135^, ^136^, ^137^ Nevertheless, most of the derivatives that showed antiviral effects were also cytotoxic. Therefore, only the compounds with sufficient selectivity between antiviral activity and cytotoxicity will be mentioned here. Xylotubercidin (**75**) possesses potent activity against herpes simplex virus (HSV‐1 and HSV‐2) (EC_50_ = 0.75 and 0.26 μM, respectively) and showed in vivo anti‐HSV effect in mice, however, the therapeutic window is rather narrow because at higher doses of xylotubercidin (**75**) its toxicity to treated animals became apparent^138^ (Fig. 19). Significant activity against hepatitis B virus (HBV) was observed for fluoroderivative **76** which is able to efficiently inhibit episomal viral replication (EC_50_ = 2.5 μM) and was well tolerated in mice.^139^, ^140^ On the other hand, compound **76** significantly affected metabolism and morphology of mitochondria and therefore it is likely to be hepatotoxic.^141^ Ethynyl derivative of 7‐deazaneplanocin A **77** showed submicromolar anti‐HBV activity (EC_50_ = 2.5 μM) and no cytotoxicity (CC_50_ > 300 μM).^125^ Although the mechanism of action of compound **77** has not been studied yet, this derivative represents an interesting lead structure as it was also active against lamivudine‐ and adefovir‐associated HBV mutants.

Several fluorinated 7‐deazapurine nucleosides showed activity against human immunodeficiency virus (HIV). Submicromolar anti‐HIV activities and no cytotoxicity were observed for derivatives **78** 143 (EC_50_ = 0.5 μM) and **79** 49 (EC_50_ = 0.71 μM) indicating that this class of nucleosides might bring more interesting anti‐HIV candidates in the future. Compound **80** bearing a 7‐(pyridine‐2‐yl)vinyl group showed promising results against influenza A virus (EC_50/H1N1 strain_ = 5.88 μM and EC_50/H3N2 strain_ = 6.95 μM) and low toxicity (CC_50_ > 100 μM).^143^ This derivative could serve as a basis for further lead optimization studies with an aim of developing novel anti‐influenza A virus agents.

Antiviral activities of 7‐deaza‐2′‐*C*‐methyladenosine (MK‐0608, **60a**) against HCV and DENV have been already mentioned. However, MK‐0608 (**60a**) was also found active against other RNA viruses such as human rhinovirus type C (HRV‐C)^144^, two viruses from *Flaviviridae* family: tick‐borne encephalitis virus (TBEV)^145^, ^146^ (EC_50_ = 1.1 μM) and recently also Zika virus (ZIKV). MK‐0608 (**60a**) showed promising anti‐ZIKV activity both in vitro^147^, ^148^ (EC_50_ = 8.92 μM) and in an in vivo mouse model.^148^ 5′‐Triphosphate form of MK‐0608 (**60a**) was identified as an inhibitor of ZIKV‐RNA‐dependent RNA polymerase (IC_50_ = 7.9 μM).^149^ As well, NITD008 (**72a**) is not only a potent anti‐dengue compound but also was shown to inhibit proliferation of enterovirus 71 (EV71) (CPE_50_ = 0.625 μM), a causative agent of Hand‐Foot‐and‐Mouth Disease^150^, TBEV (CPE EC_50_ = 0.9 μM), and other three tick‐borne viruses from *Flaviviridae* family.^151^ Co‐treatment with NITD008 (**72a**) and an anti‐inflammatory drug vorinostat (SAHA), a histone deacetylase inhibitor, was successful in a mouse model of West Nile virus (WNV) infection.^152^ Structurally similar prodrug **73** was also found to be active against many members of the *Flaviviridae* family, such as yellow fever virus and WNV.^132^

Acyclic analogues of 7‐deazapurine nucleosides and nucleotides were also screened for antiviral activities but generally these compounds showed only poor activities, especially when compared with the corresponding purine analogues.^153^, ^154^, ^155^, ^156^ Nevertheless, some acyclic analogues of 7‐bromo‐7‐deazaadenosine (**81a‐b**)^157^, ^158^, ^159^ and thiosangivamycin (**82a‐b**)^160^, ^161^, ^162^, ^163^ possess selective activity against human cytomegalovirus that is comparable or better than that of ganciclovir.

In conclusion, 7‐deazapurine nucleosides have a potential to provide new structures for development of antivirals against both DNA and RNA viruses and representatives of this class of compounds should be covered in antiviral screenings of compound libraries. 7‐Deazapurine nucleosides seem in particular promising as compounds with antiviral activities against RNA viruses from *Flaviviridae* family.

## 5 CONCLUSIONS

Replacement of N7 atom by CH in purine nucleosides is a crucial structural change, which can lead to diverse biologically active nucleoside analogues. The combination of the electronic effect of the electron‐rich pyrrole ring with the possibility of attachment of an additional substituent at position 7 does not interfere with base pairing with complementary base or recognition of the adenosine moiety by enzymes and could even increase the binding due to more efficient π–π or cation–π stacking interactions (though the only proven examples of such interactions are from our recent works on DNA polymerase incorporations of 7‐aryl‐substituted 7‐deazapurine nucloetides which were found^7^, ^8^ to be better substrates for polymerases than natural dATP or dGTP due to increased cation–π stacking in the active site of the polymerase). Due to wide range of relevant biological activities, the 7‐deazapurine moiety can be regarded as a privileged scaffold in design of antitumor or antiviral nucleosides.

Several types of deazapurine nucleosides exert promising cytostatic activities and some examples are under preclinical developments. The 7‐hetaryl‐7‐deazaadenosines (AB61 and analogues) get specifically phosphorylated in the cancer cells and get incorporated both to RNA (where they cause inhibition of the proteosynthesis) and to DNA (where they cause DNA damage). Mechanisms of the other types of cytostatic hetaryl‐substituted or thieno‐fused deazapurine nucleosides are yet under study. Many 7‐deazaadenosine derivatives are inhibitors of either human or *Mtb*‐ADK but their antimycobacterial activities were rather moderate. Many sugar‐modified 7‐deazapurine nucleosides exert antiviral activities. In particular, 2′‐*C*‐methyl‐ribonucleosides and 2′‐*C*‐methyl‐2′‐fluororibonucleosides showed promising activities against HCV and several underwent clinical trials, although none of them made it to FDA approval so far.

There is still a lot of potential in this interesting class of compounds to investigate. Most of the derivatives were derivatives of 7‐deazaadenosine, which are easier to synthesize. Much less attention was paid to derivatives of 7‐deazaguanosine, 7‐deazainosine, or 7‐deazaisoguanine. Undoubtedly, there is also a lot of space in other types of tri‐ and perhaps even tetracyclic hetero‐fused deazapurine nucleosides and in other fused heterocycles (e.g. analogues of triciribine), but their synthesis is very challenging. Combination of a modified deazapurine base with modified sugars is also an underexplored area with some potential, especially for antiviral activities against new emerging viruses. Little is known about the inhibition of protein kinases by these compounds, as well as about their interactions with adenosine receptors. 7‐Deazapurine analogues of some nucleotide cofactors could be also of interest both as drug candidates and as tools in chemical biology. We hope that this review will ignite higher attention to this important class of molecules.
